# Cross-Reactivity Antibody Response after Vaccination with Modified Live and Killed Bovine Viral Diarrhoea Virus (BVD) Vaccines

**DOI:** 10.3390/vaccines8030374

**Published:** 2020-07-11

**Authors:** Enrica Sozzi, Cecilia Righi, Massimo Boldini, Moira Bazzucchi, Giulia Pezzoni, Matteo Gradassi, Stefano Petrini, Davide Lelli, Giordano Ventura, Ilaria Pierini, Ana Moreno, Emiliana Brocchi, Antonio Lavazza, Gian Mario De Mia

**Affiliations:** 1 Istituto Zooprofilattico Sperimentale della Lombardia e dell’Emilia Romagna “Bruno Ubertini” (IZSLER), Via Antonio Bianchi 7/9, 25124 Brescia, Italy; massimo.boldini@izsler.it (M.B.); giulia.pezzoni@izsler.it (G.P.); matteo.gradassi@izsler.it (M.G.); davide.lelli@izsler.it (D.L.); giordano.ventura@izsler.it (G.V.); anamaria.morenomartin@izsler.it (A.M.); emiliana.brocchi@izsler.it (E.B.); antonio.lavazza@izsler.it (A.L.); 2 Istituto Zooprofilattico Sperimentale dell’Umbria e delle Marche “Togo Rosati” (IZSUM), Via G. Salvemini 1, 06126 Perugia, Italy; cecilia983@tiscali.it (C.R.); m.bazzucchi@izsum.it (M.B.); s.petrini@izsum.it (S.P.); i.pierini@izsum.it (I.P.); gm.demia@izsum.it (G.M.D.M.)

**Keywords:** bovine viral diarrhoea virus, subgenotypes, vaccines, virus neutralisation test

## Abstract

Pestivirus A or bovine viral diarrhoea virus (BVDV) type 1 is responsible for cosmopolitan diseases affecting cattle and other ruminants, presenting a wide range of clinical manifestations, with relevant impact on zootechnic production. The objective of the present study was to verify whether animals immunised with four commercial vaccines also developed a protective humoral immunity against other viral subgenotypes than those contained in each vaccine. Four groups of 25 bovines each were formed and vaccinated according to the manufacturer’s instructions of the commercial vaccines. On sera collected 28 days after the last vaccination, virus neutralisation tests (VNT) were performed using homologous and heterologous viruses and enzyme-linked immunosorbent assay (ELISA) methods. Finally, the VNT results were comparatively evaluated through a statistical analysis. Serological results highlighted that, although with a different degree of efficiency, the four vaccines resulted in not developing a solid antibody-mediated cross-immunity against all the strains used.

## 1. Introduction

Bovine viral diarrhoea virus (BVDV) is a widespread and economically important cattle pathogen associated with a variety of clinical syndromes, including subclinical infections, respiratory disease, intestinal and reproductive disorders, immunosuppression, persistent infection, and mucosal disease. The virus belongs to the *Pestivirus* genus of the *Flaviviridae* family. Currently, the International Committee on Taxonomy of Viruses (ICTV) recognises 11 Pestivirus species named from A to K [1]. The three former species BVDV-1, BVDV-2 and the putative third bovine species, referred to as HoBi-like pestivirus, are now named respectively Pestivirus A, Pestivirus B, and Pestivirus H. Phylogenetic analysis of these three bovine pestiviruses has further classified them into subgroups (subgenotypes) and at least 21 BVDV-1 (1a–1u), four BVDV-2 and four HoBi-like subgroups [2,3] have been identified. The extensive genetic diversity of BVDV reflected by the number of detected subgenotypes has been described also in Italy [4]. In particular, the presence of at least 14 different BVDV-1 subgenotypes was demonstrated, according to four distinct distribution patterns [5]. BVDV-2 remains a sporadic event, while the presence of HoBi-like pestivirus was reported in some farms in southern Italy [6]. Due to the high economic losses caused by this virus, an awareness of BVD and the need for BVDV eradication have increased during the last 20 years. Control of BVDV involves removing persistently infected (PI) animals from the herd, ensuring biosecurity level of the farms and vaccination of susceptible animals [7]. Vaccination with either live or killed BVDV vaccines is routinely practiced in attempts to control the disease. The most important outcome of BVDV vaccination is to protect heifers and cows against transplacental infection that leads to the generation of persistently infected animals. However, vaccination of calves to provide protection from clinical infection is also necessary to minimise the economic losses due to BVDV infection. Several vaccines for BVDV are available in Italy, either conventional inactivated and adjuvanted vaccines or modified live virus (MLV) vaccines with reduced virulence. Previous studies have examined the impact of BVDV vaccination on the results of antibody testing under controlled conditions [8,9,10]. In fact, neutralising antibodies to BVDV have been widely used as an important parameter to evaluate the immune responses for both vaccination and natural infection [11,12,13]. Moreover, enzyme-linked immunosorbent assays (ELISAs) for the detection of antibodies versus specific viral proteins provide valuable information about important aspects of the immune response. Antigenic differences in field strains can affect not only diagnostic analysis but also protection induced by vaccines against infection. In fact, it was demonstrated that previous infections or vaccination with a pre-defined BVDV strain may not create sufficient protection against different strains [14,15]. Traditional commercial vaccines in Italy contain only a few subgenotypes (mostly BVDV-1a and BVDV-1b), and it remains unclear whether these vaccines are able to fully protect against circulating subgenotypes other than those contained in them. Thus, the objective of the present study was to verify whether animals immunised with four commercial vaccines were also able to develop a protective humoral immunity against viral subgenotypes other than those contained in each vaccine. Therefore, killed and MLV BVDV vaccines were administered to BVDV seronegative heifers. Specific humoral and neutralising responses to different subgenotypes of BVDV type 1 were tested before vaccination and at 28 days after vaccination.

## 2. Materials and Methods 

### 2.1. Animals and Vaccination Design 

In a BVDV controlled herd, 100 one-year-old heifers, previously tested antigen and antibody negative, were randomised and divided into four groups each consisting of 25 animals. The animals of each group received one of the following commercially available BVDV vaccines, according to the manufacturer’s instructions:

(a) Bovilis^®^ BVD-MD (Msd Animal Health, Madison, NJ, USA)
(b) Rispoval^®^ D-BVD (Pfizer, New York, NY, USA)
(c) Mucosiffa^®^ (Merial, Lyon, France)
(d) Bovela^®^ (Boehringer Ingelheim, Ingelheim, Germany)

The live modified and inactivated vaccines were composed of BVDV genotype type 1 strains, as described in Table 1. The table also reports the vaccination design. 

Bovela^®^ vaccine is a live double deleted BVDV vaccine containing both BVDV type 1 and 2 strains, however, this study did not take into account the immune response to the BVDV-2 genotype.

Animals from each group were housed together in a separate stable from the others for the entire duration of the study. Serum samples from all animals of the four groups were collected twice respectively on the day of vaccination (T0) and 28 days after vaccination or after booster vaccination, if required (T1). 

Both vaccination and serum sampling were performed by vet practitioners who applied good medical practice in agreement with national regulations on animal welfare.

### 2.2. Virus Neutralisation Test (VNT)

(VNT) was performed according to the protocol described in the World Organisation for Animal Health (OIE) manual [16] by employing five test viruses belonging to three different BVDV-1 subgenotypes (BVDV-1a, -1b, -1e). These viruses were of the cytopathic biotype and were selected according to their phylogenetic analysis (Table 2), i.e., strains homologous to the vaccine strains and those antigenically more distant strain were chosen. Different genomic regions have, so far, been used for genotyping and classification of BVDVs; nevertheless, partial 5’-UTR sequences have been most frequently used for phylogenetic analysis and genotyping, followed by N^pro^ and, to a lesser extent, the E2 coding sequence [3]. Accordingly, the analysis to establish the subgenotype of the test viruses employed in our study has been performed by analysis of 5′-UTR genomic region.

The viruses used for VNTs were propagated in the Madin–Darby bovine kidney (MDBK) cell line, grown in minimal essential medium (MEM) supplemented with (10%) foetal bovine serum. The foetal bovine serum and cell line were both found to be free of BVDV antigen and antibodies when tested before use. Briefly, serial 2-fold dilutions of heat-inactivated sera were made in a volume of 0.025 mL in a microplate with the cell culture medium as diluent, and an equal volume of the viral working suspension containing 100 TCID_50_ (50%) tissue culture infective dose was added and incubated at 37 °C and 5% CO_2_ for 1 h. Control positive and negative sera were also included in each batch of tests. Then, 0.050 mL of MDBK cell suspension (about 1.5 × 10^5^ cells/mL) was added into all wells of the microplate and incubated at 37 °C and 5% CO_2_. After 4–5 days of incubation, cells were observed using microscopy. The highest serum dilution that inhibited the virus propagation in 50% of the wells tested in duplicates was considered to be the virus neutralisation titre. For the VNT to be valid, the titration of the viral working suspension had to yield a virus concentration of 30–300 TCID_50_/0.025 mL.

### 2.3. Indirect ELISA Tests

Sera of all vaccinated animals were tested for the presence of specific antibodies using two indirect ELISA tests, generated at IZSLER, for the detection of antibodies, one against the immunogenic and highly conserved non-structural protein 3 (NS3) of pestivirus and the other against the envelope glycoprotein E2 of BVDV, known to elicit neutralising antibodies. Both tests are regularly used in the diagnostic routine of IZSLER. These ELISAs use both recombinant antigens, respectively NS3 and E2, derived from the reference strain NADL and expressed in a baculovirus system [17]. The recombinant proteins are fixed onto the ELISA solid-phase by means of specific capture monoclonal antibodies (MAbs) [18]. Briefly, the MAbs 3H4 and 3H1 were adsorbed onto a Nunc® MaxiSorp™ microplate (Thermo Fisher Scientific Inc. Waltham, MA USA) at a saturating concentration to trap, respectively, the recombinant NS3 and E2 antigens. Sera diluted 1/50 are dispensed in duplicate wells, one containing antigen and the other not (only buffer). After incubation and washing, a pre-determined optimal dilution of the proprietary MAb 1G10 against ruminant immunoglobulin G (IgG), peroxidase-conjugated, was delivered. After the development of the reaction, results were reported as S/P values, calculated as the ratio between the net optical density (OD) of the sample (obtained by subtracting the OD of the control well without antigen from the OD of the well with antigen) and the net OD of a positive control serum tested in each plate, with the positive cut-off value for serum samples set at 0.1. 

### 2.4. Statistical Analysis

To evaluate the presence of statistically significant differences between the results of the VNT with the five viral strains, the Wilcoxon test was used (considering a *p*-value less than or equal to 0.05 significant). The data analysis was carried out using the Stata 11.2 software (StataCorp LLC, College Station, TX, USA).

## 3. Results

The serological investigation conducted at T0 confirmed the absence of viral circulation. In fact, all animals were seronegative. Animals remained healthy throughout the study. The evaluation of the results obtained was carried out both through a descriptive analysis and through an inferential analysis. 

### 3.1. Virus Neutralisation Antibody Titres

First, it was evaluated which neutralising antibody titre can be considered protective. There are few data in the literature in this regard; however, Ridpath J.F. et al. [19] set the minimum cross-protection threshold between BVDV-1 and BVDV-2 in 1/16. On this basis, we considered antibody titres ≥1/20 as indicative of cross-protection in our study.

**Figure 1:**
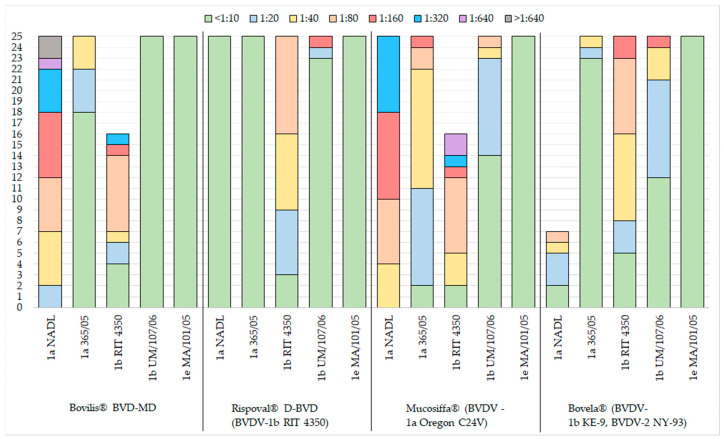
Results for distribution of inoculated vaccine and virus neutralisation in cattle for the 5 viral strains employed.

The neutralising antibody titres among the studied BVDV-1 subgenotypes are presented in Figure 1.

Therefore, evaluating all virus-neutralising titres over the fixed threshold (≥1/20), we noted that, although with a different degree of efficiency, the four vaccines used did not elicit cross-neutralising antibody against all the subgenotypes employed. In detail:

- Bovilis^®^ BVD-MD (BVDV-1a) induced titres ≥1/20 against BVDV-1a NADL (25/25) and in most animals, also against the heterologous subgenotype BVDV-1b, RIT 4350 (21/25), but elicited a poor immunity versus the homologous genotype BVDV-1a 365/05;
- Rispoval^®^ D-BVD (BVDV-1b) induced consistent neutralising antibodies only against the homologous strain (22/25);
- Mucosiffa^®^ (BVDV-1a) induced neutralising antibodies with titres ≥1/20 against the two strains of the homologous subgenotype BVDV-1a NADL (25/25) and BVDV-1a 365/05 (23/25), but also against the heterologous subgenotype strain BVDV-1b RIT 4350 (23/25);
- Bovela^®^ (BVDV-1b and BVDV-2) induced neutralising antibodies with titres ≥1/20 against the two strains of the homologous subgenotype BVDV-1b RIT 4350 (20/25) and BVDV-1b UM/107/06 (13/25), with poor cross-reaction against the two strains of the heterologous subgenotype BVDV-1a.

The results show the complete absence of virus-neutralising antibodies against BVDV-1e in all vaccinated animals, independently on the vaccine used. Furthermore, each vaccine induced neutralising antibodies against at least one of the two strains of the homologous subgenotype, and, in two cases (Bovilis^®^ BVD-MD and Mucosiffa^®^), also against at least one strain of the heterologous subgenotype (Figure 1). 

### 3.2. Indirect ELISA Tests

**Figure 2:**
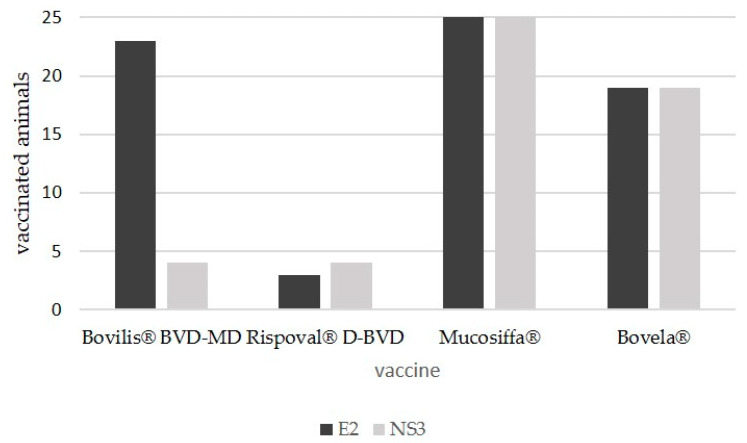
Frequency distribution of vaccinated animals tested positive for bovine viral diarrhoea virus (BVDV) structural (E2) and non-structural (NS3) proteins.

The serological investigations by the two indirect ELISAs for the detection of antibodies, respectively, against the BVDV NS3 and E2 proteins, provided the following results (Figure 2):

- Bovilis^®^ BVD-MD (BVDV-1a): 23 animals out of 25 (23/25) developed antibodies against structural E2 protein and only 4 against NS3 (4/25);
- Rispoval^®^ D-BVD (BVDV-1b): 3 animals out of 25 (3/25) developed antibodies against structural E2 protein and only 4 against NS3 (4/25);
- Mucosiffa^®^ (BVDV-1a): all animals (25/25) developed antibodies against both structural E2 and NS3 proteins;
- Bovela^®^ (BVDV-1b and BVDV-2): 19 animals of 25 (19/25) developed antibodies against both structural E2 and NS3 proteins.

### 3.3. Statistical Analysis 

As for the inferential analysis, post hoc tests were used to compare the antibody responses toward the strains used in the VNT (Table 3). 

Specifically, the titres obtained with the homologous strain when available or, alternatively, those obtained with the strain that could be defined as the “proxy variable” were compared, two by two, with each of the other viruses used. The results support what emerged in part during the descriptive analysis, pointing out significant differences between the homologous genotype (or proxy variant) and almost all the other strains, except for one case (BVDV-1a NADL vs. BVDV-1b RIT 4350, in the group vaccinated with Mucosiffa^®^). Further evidence is the difference found between isolates of the same subgenotype, for example, NADL vs. 365/05 (both BVDV-1a) and RIT 4350 vs. UM/107/06 (both BVDV-1b), which also emerged in descriptive analysis. In fact, it is well known that viruses homogeneous from an antigenic point of view are not necessarily included within the same subgenotype [20]. Indeed, the criteria by which membership of one subgenotype is assigned rather than another does not necessarily correlate with the antigenic characteristics. The subgenotype is assigned on the basis of the sequence identity percentage in regions of the genome (5’-UTR and/or N^pro^) where the most viral immunogenic proteins do not reside (mostly located in regions E2 and NS 2–3). This explains why even significant antigenic differences could exist within the same subgenotype.

## 4. Discussion

BVD is a widely distributed viral infection in cattle populations and causes economic losses every year. Therefore, many countries have placed a large emphasis on the control of the disease and on its prevention. The phylogenetic and neutralisation analysis of BVD viruses have revealed many subgenotypes within three main genotypes. It remains unclear whether there is a relationship between the clinical findings and genetic subgenotypes of BVDV; however, there some results indicate discrepancies among BVDV subgenotypes that may impact the disease prevention and diagnosis [21,22]. Hence, a demonstration of the serological relatedness among the BVDV subgenotypes may be crucial for the diagnosis, prevention and control of the infection. Previous studies [23] have demonstrated antigenic variations between the BVD-1 and BVD-2 strains, which may lead to a poor vaccine cross-protection against the other strains. In fact, viruses homogeneous from an antigenic point of view are not necessarily included within the same subgenotype [20]. Indeed, the criteria by which membership of one subgenotype is assigned rather than another does not necessarily correlate with the antigenic characteristics and this explains why even significant antigenic differences could exist within the same subgenotype. A further evaluation of the antigenic differences among BVDV-1 subgenotypes, which may affect not only diagnostic analysis but also protection against infection, has been described in previous studies by cross-neutralization assays and by calculating the coefficient of antigenic similarity (*R*) [20,24,25]. However, to exactly define the coefficient of antigenic similarity (*R*), it is essential to have available both the viruses (in this case the vaccinal strains) and accompanied homologous antisera. 

Antibodies induced by vaccination may be responsible for protective humoral immunity since previous studies have shown that passive transfer of BVDV-specific antibodies can confer protection against systemic challenge with BVDV. Beer et al. [26] described a ≥1:512 neutralising antibody titre was required for marked protection against BVDV infection, although a viral neutralisation titre of 1:256 was found to be critical for the prevention of clinical symptoms [27]. The possibility of a high level of serological differences has also been suggested by different authors, and the relationship between VNTs and protection remains controversial [20,28]. Hence, the aim of this work was to verify whether animals immunised with four commercial vaccines would develop a solid antibody-mediated cross-immunity also against viral subgenotypes different than those contained in each vaccine. 

To establish this, an in vitro study was carried out by setting up a series of virus neutralisation tests with five different BVDV-1 subgenotypes to evaluate the degree of cross-reactivity of each group of animals against these viral strains. For this purpose, two BVDV-1a strains were selected, two BVDV-1b (one of which is the same strain used for the preparation of one of the vaccines employed) and one BVDV-1e strain, which was chosen because the prediction of the antigenic sites carried out in silico by sequence analysis indicated a marked antigenic difference compared to the other subgenotypes. The obtained results showed that the virus-neutralising antibody response induced by the vaccines was (i) unable to neutralise all tested viral strains, irrespective of the subgenotype homology; (ii) completely absent against BVDV-1e, thus indicating the existence of significantly different epitopes effective for the neutralisation between BVDV-1e and the other tested viruses; (iii) each vaccine induced neutralising antibodies against at least one of the two strains of the homologous subgenotype; (iv) Bovilis^®^ BVD-MD and Mucosiffa^®^ were also able to induce detectable levels of cross-reacting antibodies against at least one other subgenotype. Interestingly, the differences between BVDV-1a and BVDV-1b found in this study were similar to those reported in previous studies [20,29,30]. Other studies showed a low level of antibody response to the BVDV-1b subgenotype by BVDV-1a vaccines [31] and some insufficient responses to protect against BVDV-1b infections [15,30]. Bachofen et al. [25] reported variable differences in the R values between field isolates belonging to different BVDV-1 subgenotypes. In particular, values indicative of significative antigenic difference (*R* ≤ 25), were detected between subgenotype 1e and both 1a and 1b, in accordance with the results obtained in our study, although by using a different methodological approach. 

The ELISA tests used in the diagnostic routine detected the antibody response induced by vaccines, but with some peculiarities: (i) Bovilis^®^ BVD-MD, induced the production of antibodies against structural E2 protein, but not against NS3 protein. In fact, studies have shown that some but not all inactivated BVDV vaccines do not induce detectable antibodies against the NS3 proteins [8,32] and they can be considered, at least under certain conditions, as marker vaccines able to distinguish the vaccinated animals from those that have contracted the infection [9]; (ii) Despite the negativity toward the BVDV structural and non-structural proteins, the Rispoval^®^ D-BVD vaccine is able to stimulate a significant production of neutralising antibodies toward the homologous strain, indicating that all the vaccinated animals of this group were successfully immunised and mounted a detectable humoral immune response. NS3 is highly immunogenic and prerequisite for the BVDV replication complex; thus, the detection of NS3 antibodies after vaccination is a marker for viral replication. Interestingly, none of the animals vaccinated with Rispoval^®^ D-BVD developed detectable NS3-specific antibodies. Probably, the vaccine strain of Rispoval^®^ D-BVD involves viremic levels so low that it does not induce production of detectable antibodies against the non-structural proteins of the virus by ELISA tests. The majority of the immune response is also directed toward viral protein E2. It is possible that the antibody specificity to E2 may depend on the genomic origin of E2 recombinant antigen used in the ELISA assay (BVDV-1a NADL strain). Therefore, the antigenic differences between subgenotypes BVDV-1a NADL strain and 1b RIT4350 strain may likely explain such negative results against the BVDV structural proteins. Indeed, variations of E2 in field pestivirus strains may influence the reaction patterns and cross-neutralization with sera from heterologous strains, but such variations cannot be highlighted through phylogenetic analysis but rather through in silico prediction of the antigenic epitopes that might have a potential for serologically discrimination.

Finally, the results showed that the vaccines considered here, which are among those occupying the largest market shares in our country, are unable to confer a solid cross-immunity against any subgenotype of BVDV-1 which may be responsible of infection. This is true for the five strains used in this study, but it can be reasonably assumed that the scenario, in reality, is even worse, considering that, in Italy, more than 14 different genotypes are circulating. Certainly, the absence of national control or eradication plans, the dense network of commercial exchanges and the consequent movement of animals contribute to a major exposure to the risk of BVDV introduction on the farm as well as the entry of new viral variants. However, what emerges from the present study adds a further element of risk linked to insufficient vaccination-induced protection, which explains, at least in part, why this disease is so widespread despite the extensive use of vaccination practice. Finally, the authors underline that the present research did not aim to carry out a comparative evaluation between different immunising products, nor to define efficacy rankings based on the results acquired.

## 5. Conclusions

Our results confirmed that between different subgenotypes of BVDV, in particular, between 1a and 1b, and the cross reactivity in virus neutralisation assay could be significantly low. In Italy, most used vaccines contain the BVDV 1a strain, although the BVDV 1b strain is the most commonly detected in field; therefore, the choice of a vaccine should also be based on these epidemiological data. There are BVDV control and eradication programmes currently administered in different countries. Because some of these programmes are primarily based on vaccination strategies, the serological discrepancies between the vaccines and field strains should be carefully considered and monitored over time.

## Figures and Tables

**Table 1:** Characteristics of vaccinal strains for commercial vaccines and vaccination design.

Vaccine	Strain	Subgenotype	Inactivated/Live Modified	Vaccination Schedule
Bovilis^®^ BVD-MD	C86	1a	Inactivated	Twice at an interval of 1 month
Rispoval^®^ D-BVD	RIT4350	1b	Live modified	Twice at an interval of 1 month
Mucosiffa^®^	C24V	1a	Live modified	Once
Bovela^®^	KE-9	1b	Live modified	Once

**Table 2:** Background details of the viral strains used in the virus neutralisation assays.

Strain	Genotype	Subgenotype	Biotype
NADL	BVDV-1	1a	Cp *
365/05	BVDV-1	1a	Cp
RIT4350	BVDV-1	1b	Cp
UM/107/06	BVDV-1	1b	Cp
MA/101/05	BVDV-1	1e	Cp

* Cp: cytopathic.

**Table 3:** Wilcoxon test results (*p*-value), through Napierian logarithm, to evaluate the difference between post-vaccination antibody titres against the homologous virus (or the proxy variable) and other selected test viruses; (*) statistical significance *p* < 0.05.

Vaccine	Reference Strain	BVDV Strains Used for the SN Test/*p*-Value (*)			
Bovilis^®^ BVD-MD		1a 365/05	1b RIT 4350	1b UM/107/06	1e MA/101/05
	1a NADL	<0.0001 (*)	0.0011	<0.0001	<0.0001
Rispoval^®^ D-BVD		1a NADL	1a 365/05	1b UM/107/06	1e MA/101/05
	1b RIT 4350	<0.0001	<0.0001	<0.0001	<0.0001
Mucosiffa^®^		1a 365/05	1b RIT 4350	1b UM/107/06	1e MA/101/05
	1a NADL	<0.0001	0.3545	<0.0001	<0.0001
Bovela^®^		1a NADL	1a 365/05	1b UM/107/06	1e MA/101/05
	1b RIT 4350	<0.0001	<0.0001	0.0004	<0.0001
